# Effects of different feeder layers on culture of bovine embryonic stem cell-like cells in vitro

**DOI:** 10.1007/s10616-013-9653-4

**Published:** 2014-05-08

**Authors:** Shan Cong, Guifang Cao, Dongjun Liu

**Affiliations:** 1 Department of Veterinary, Inner Mongolia Agricultural University, Hohhot, 010018 MO People’s Republic of China; 2 Key Laboratory of Mammalian Reproductive Biology and Biotechnology, Ministry of Education, Inner Mongolia University, Hohhot, 010021 MO People’s Republic of China

**Keywords:** Bovine embryonic stem cell-like cells, Feeder layer, Pluripotency-related gene, Real-time quantitative PCR

## Abstract

To find a suitable feeder layer is important for successful culture conditions of bovine embryonic stem cell-like cells. In this study, expression of pluripotency-related genes OCT4, SOX2 and NANOG in bovine embryonic stem cell-like cells on mouse embryonic fibroblast feeder layers at 1–5 passages were monitored in order to identify the possible reason that bovine embryonic stem cell-like cells could not continue growth and passage. Here, we developed two novel feeder layers, mixed embryonic fibroblast feeder layers of mouse and bovine embryonic fibroblast at different ratios and sources including mouse fibroblast cell lines. The bovine embryonic stem cell-like cells generated in our study displayed typical stem cell morphology and expressed specific markers such as OCT4, stage-specific embryonic antigen 1 and 4, alkaline phosphatase, SOX2, and NANOG mRNA levels. When feeder layers and cell growth factors were removed, the bovine embryonic stem cell-like cells formed embryoid bodies in a suspension culture. Furthermore, we compared the expression of the pluripotent markers during bovine embryonic stem cell-like cell in culture on mixed embryonic fibroblast feeder layers, including mouse fibroblast cell lines feeder layers and mouse embryonic fibroblast feeder layers by real-time quantitative polymerase chain reaction. Results suggested that mixed embryonic fibroblast and sources including mouse fibroblast cell lines feeder layers were more suitable for long-term culture and growth of bovine embryonic stem cell-like cells than mouse embryonic fibroblast feeder layers. The findings may provide useful experimental data for the establishment of an appropriate culture system for bovine embryonic stem cell lines.

## Introduction

Embryonic stem cells have been derived from the inner cell masses (ICM) of blastocysts in many species. They are capable of unlimited undifferentiated proliferation on feeder layers and remain karyotypically normal and phenotypically stable. In addition, embryonic stem cells have the ability to differentiate into a wide variety of cell types in vivo and in vitro (Christoforou et al. 2010; Zeevi-Levin et al. 2010; Evans and Kaufman 1981). Embryonic stem cell lines have been established from pigs, humans, sheep, mice and rabbits (Piedrahita et al. 1990; Thomson et al. 1998; Wells et al. 1997; Evans and Kaufman 1981; Graves and Moreadith 1993). To date, suitable culture conditions for preventing spontaneous differentiation of bovine embryonic stem cells have not been established despite the extensive efforts devoted to the generation of stable bovine embryonic stem cell or bovine embryonic stem cell-like cell lines (Cibelli et al. 1998; Iwasaki et al. 2000; Mitalipova et al. 2001; Saito et al. 2003; Stice et al. 1996; Wang et al. 2005; Strelchenko 1996; Gong et al. 2010). Because of their potential use for targeted gene manipulation, isolation of bovine embryonic stem cells in livestock species could have numerous agricultural, biomedical and pharmaceutical applications (Anand et al. 2011). Thus, improvements are needed in order to maintain the undifferentiated pluripotent state of bovine embryonic stem cell or bovine embryonic stem cell-like cell lines.

Previous studies attempted to use different kinds of feeder cells to support embryonic stem cell growth and prevent cell differentiation (Piedrahita et al. 1990; Smith and Hooper 1987). Researchers often traditionally use single mouse embryonic fibroblast or bovine embryonic fibroblast as feeder layers to support bovine embryonic stem cell growth (Seizo et al. 1998; Muñoz et al. 2008). Several studies suggest that heterologous murine embryonic fibroblast feeder layers are better than homologous feeder layers for most embryonic stem cells (Lim and Bodnar 2002). However, murine embryonic fibroblast feeder layers tend to lose capacity to support proliferation of bovine embryonic stem cell-like cells with increasing passages, and the reason for this remains unclear. Therefore, it is necessary to find a more suitable feeder layer system for bovine embryonic stem cell-like cell growth. The mixed embryonic fibroblast feeder layers were produced from mouse and bovine embryonic fibroblast at different ratios. The sources including mouse fibroblast cell line (STO) were derived from mouse embryonic fibroblasts, were resistant to 6-thioguanine and ouabain, and were sensitive to hypoxanthine guanine phosphoribosyl transferase (HPRT) and hypoxanthine, aminopterin, and thymidine (HAT), as well as negative for mouse poxvirus (Park et al. 2003). Yet, few researchers have compared the effects of using different types of feeder layers for the culture of bovine embryonic stem cell-like cells.

Recent findings having identified the transcription factors involved in the regulation of pluripotency and self-renewal in embryonic stem cells may provide keys that enable the derivation of embryonic stem cells in livestock species. It is believed that the transcription factors OCT4, SOX2, and NANOG play significant roles in maintaining pluripotency and self-renewal of embryonic stem cells (Sun et al. 2006; Huang et al. 2010). The correct expression level of OCT4, SOX2, and NANOG is considered a key marker for embryonic stem cells because it is essential for maintenance of an undifferentiated state, when the expression of OCT4, SOX2, and NANOG have decreased or disappeared, the stem cells have usually differentiated (Avilion et al. 2003; Pesce and Schöler 2001; Miyanari and Torres-Padilla 2012). However, the expression of the maker genes in the bovine embryonic stem cells needs more research.

In this study, we compared the culture effects of feeder layers composed of different cell types on bovine embryonic stem cell-like cells. Our findings may provide useful data for the establishment of authentic bovine embryonic stem cells lines.

## Materials and methods

### Preparation of feeder cell layers

Irradiated primary murine embryonic fibroblast cells (Key Laboratory of Mammalian Reproductive Biology and Biotechnology Ministry of Education, Huhhot, China) were used as described previously (Reubinoff et al. 2000). For the preparation of bovine embryonic fibroblast, bovine fetuses at 4–6 weeks were washed with warm saline. After removal of the head, limbs and innards, the carcasses were cut into pieces. The tissue pellets were placed on 100 mm dishes (Corning Life Sciences, Corning, NY, USA) and incubated for 30 min at 37 °C under an atmosphere of 5 % CO_2_. Bovine embryonic fibroblast growth medium (Cell Applications, San Diego, CA, USA) was added for further culture. The medium was changed every 2 days and passaged every 4–6 days. The bovine embryonic fibroblast at the fifth or sixth passage were inactivated in a medium DMEM/F12 (Gibco, Grand Island, NY, USA) containing 10 ng/ml mitomycin-C (Sigma, St. Louis, MO, USA) for 2.5–3 h, washed three times with Ca^2+^ and Mg^2+^ free phosphate buffered saline [PBS(−)] and then treated with 0.25 % trypsin-0.02 % EDTA solution. The trypsinized cells were harvested by centrifugation at 500*g*. For preparation of mixed feeder layers with murine embryonic fibroblast and bovine embryonic fibroblast feeder layers, the two fibroblasts were, respectively, counted and mixed together according to the ratios of 0:1, 1:1, 1:2, 2:1 and 1:0. The mixture was then inoculated based on a density of 2–2.5 × 10^4^ cells/cm^2^, wells in a four-well plate coated with 0.1 % gelatin (Sigma). Both murine embryonic fibroblast and bovine embryonic fibroblast were plated 1 day before inner cell masses were seeded (Muñoz et al. 2008; Roach et al. 2006; Bettiol et al. 2007; Vitezslav et al. 2006; Li et al. 2004). The bovine embryonic stem cell-like cells were cultured on mixed feeder layers using bovine embryonic stem cell-like basic culture medium which consisted of 90 % DMEM/F12 (Gibco) supplemented with 10 % fetal bovine serum (Gibco), 0.1 mM β-mercaptoethanol (Chemicon, Temecula, CA, USA), 0.1 mM nonessential aminoacids (Chemicon), 100 IU/ml penicillin (Gibco), 0.05 mg/ml streptomycin (Gibco), 20 ng/ml leukemia inhibitory factor (LIF) (Millipore, Billerica, MA, USA) and 10 ng/ml basic fibroblast growth factor (bFGF) (Promega, Madison, WI, USA). The STO cell line was purchased from the Chinese Academy of Sciences and cultured in Dulbecco modified Eagle medium (DMEM) high-glucose (Gibco) supplemented with 10 % fetal bovine serum (Gibco). Cells were treated with 10 mg/ml mitomycin C (Sigma) for 1.5 h.

The mitomycin C treated STO cells were extensively washed in PBS and replated at 2–2.5 × 10^4 ^cells/cm^2^ in a four-well plate coated with 0.1 % gelatin (Sigma). STO cells were plated 1 day before inner cell masses were seeded. Basic culture medium of bovine embryonic stem cell-like cells was as described above.

### In vitro fertilization (IVF) derived embryos

Oocytes collected from cattle ovaries at an abattoir were cultured in TCM 199 medium (Gibco, Gaithersburg, MD, USA) supplemented with 10 mmol/l Hepes (Sigma), 1 μg/ml estradiol (Sigma) and 10 μg/ml follicle stimulating hormone (Kawazaki, Tokyo, Japan) for 22–24 h. The mature oocytes were fertilized in vitro using commercial bovine semen (Inner Mongolia Livestock Breeding and Improving Center, 87 Huhhot, China) as described previously (Tervit et al. 1972). The zygotes were cultured in an in vitro production system using SOFaa medium (Key Laboratory of Mammalian Reproductive Biology and Biotechnology Ministry of Education, Huhhot, China) supplemented with 10 % fetal bovine serum (Gibco). The blastocysts were hatched in 8–11 days. The hatched blastocysts were cultured on feeder layers with culture medium which consisted of 90 % DMEM/F12 (Gibco) supplemented with 10 % fetal bovine serum (Gibco), 0.1 mM β-mercaptoethanol (Chemicon), 0.1 mM nonessential amino acids, 100 IU/ml penicillin, 0.05 mg/ml streptomycin, 20 ng/ml LIF, 10 ng/ml bFGF. After the hatched blastocysts attached on the feeder layers, inner cell masses that grew well were used for passaging.

### Mechanical passaging method of bovine embryonic stem cell-like cells

Embryonic stem cell-like colonies grew well after the ICMs had been cultured on feeder layers for 3–5 days. For the mechanical passaging, the colonies were dissected in a microdrop under a microscope by repeated pipetting using a micropipette. The disaggregated colony cells were reseeded onto new feeder layers. The cells were cultured in basic culture medium of bovine embryonic stem cell-like cell.

### Alkaline phosphatase staining

Alkaline phosphatase (ALP) activity was determined as previously described (Takahashi and Yamanaka 2006). Briefly, culture medium was removed from the plates and bovine embryonic stem cell-like cells were fixed with 4 % paraformaldehyde for 20 min. Fixed cells were washed twice with PBS and stained in 200 μg/ml naphtol AS-MX phosphate (Sigma) and 1 mg/ml Fast Red TR salt (Sigma) in 100 mM Tris buffer, pH 8.2 for 30 min at room temperature. Staining was terminated by washing cultures in PBS. Positive ALP staining was characterized by red color.

### Immunocytochemical staining

Before preparation of feeder layers, the sterile coverslip was placed in the well in a four-well plate. Bovine embryonic stem cell-like cells grown on coverslips were washed, fixed with 4 % paraformaldehyde, permeabilized with PBS containing 0.1 % (vol/vol) Triton X-100, and incubated sequentially with 0.4 % bovine serum albumin (Sigma) in PBS. The cells were pretreated with 10 % normal goat serum (Hyclone, Logan, UT, USA)-PBS for 30 min. They were then incubated with monoclonal antibodies against OCT4, stage-specific embryonic antigen 1 and 4 (SSEA-1 and SSEA-4), followed by secondary antibodies (Biosynthesis Biotechnolgy Co. Ltd., Beijing, China, bsf-0368G), using an embryonic stem cell marker sample kit (Chemicon, Temecula, CA, USA) according to the protocol supplied by the manufacturer. Immunofluorescence images were observed under a fluorescence microscope.

### In vitro differentiation

To induce embryoid body (EB) formation, bovine embryonic stem cell-like colonies were cut into small pieces, then cultured in 35-mm feeder layer-free petri dish, cultured with LIF and bFGF-free embryonic stem cell suspension (medium: 90 % DMEM/F12 (Gibco) supplemented with 10 % fetal bovine serum (Gibco), 0.1 mM β-mercaptoethanol (Chemicon), 0.1 mM nonessential amino acids, 100 IU/ml penicillin, 0.05 mg/ml streptomycin) for 7–10 days, and observed under microscope.

### OCT4, SOX2 and NANOG expression

Expression of pluripotent factors was also detected by reverse transcriptase-polymerase chain reaction (RT-PCR). Total RNA was prepared using a QIAGEN RNeasy kit (QIAGEN, Valencia, CA, USA). Standard reverse-transcription reactions were performed with 500 ng of total RNA using random hexamers and AMV reverse transcriptase (Roche Molecular Biochemicals, Mannheim, Germany). The PCR was carried out with 2 ml of cDNA template, 1 ml of 10 mM dNTP mixtures, 10 pmol of OCT4, SOX2 and NANOG. The OCT4, SOX2 and NANOG RNA transcripts were amplified using 5 min for denaturation at 94 °C, followed by 40 cycles at 94 °C for 30 s, with the final extension at 72 °C for 10 min, 62 °C for 30 s, and 72 °C for 30 s in a GeneAmp 9600 (Perkin-Elmer, Irvine, CA, USA). As a loading control, the same amounts of cDNA template were amplified using GAPDH. Products were analyzed on 1.5 % agarose gel and visualized by ethidium bromide staining.

Primer sequences were as follows: GAPDH 5′ untranslated region (5′-CAAGTTCAACGGCACAGTCA-3′) and 3′ untranslated region (5′-CCACCACATACTCAGCACCAG-3′); OCT4 5′ untranslated region (5′-AGGTGTTCAGCCAAACGACTATC-3′) and 3′ untranslated region (5′-TCAGCTTCCTCCACCCACTTC-3′); SOX2 5′ untranslated region (5′-TCAGATGCAGCCCATGCAC-3′) and 3′ untranslated region (5′-GGTGCCCTGCTGAGAATAGGAC-3′); NANOG 5′ untranslated region (5′-CATCTGCTGACACCCTCGACA-3′) and 3′ untranslated region (5′-GGGTCTGCGAGAACACAGTTCTAA-3′).

Amplifications yielded products of 106 bp (OCT-4), 195 bp (NANOG), 121 bp (SOX2), and 128 bp (GAPDH).

### Real-time quantitative PCR (RT-qPCR)

The bovine embryonic stem cell-like colonies were processed using a RNAiso Reagent from TaKaRa (Shiga, Japan) using the manufacturer’s recommendations. To completely remove genomic DNA contamination from the RNA extraction, we performed the in-column DNAse I optional step using amplification grade DNAse I (Invitrogen™). A final volume of 15 μl of RNAse free water was used in RNA elution. Only RNA samples with a 260/280 nm wavelength ratio above 2.00 were used for RT-qPCR assays. Approximately 650 ng of RNA from each sample was used in subsequent reverse transcriptase reactions. Complementary DNA was synthesized using the PrimeScript™ RT reagent Kit (Perfect Real Time, TaKaRa) according to the manufacturer’s recommendations. Synthesis of the first strand was carried out at 37 °C for 15 min followed by a 15 s step at 85 °C for inactivation of reverse transcriptase. Primers were designed using the Primer3 webtool with settings to generate primers with a melting temperature of ~60 °C. Due to the limitations of extension time in quantitative polymerase chain reactions (qPCR), primers were designed to amplify less than 200 bp of sequence when possible.

All qPCR experiments were performed using the SYBR Premix 2× Ex Taq (TaKaRa). Reaction volumes were reduced to 20 μl. A 7300 Real Time PCR System (Applied Biosystems, Foster City, CA, USA) was used to quantify reactions. This was followed by one 95 °C denaturation for 30 s. Temperature cycling consisted of the following: 35 cycles of 95 °C for 5 s then 60 °C for 30 s. Melt curves (to determine if there were multiple PCR amplicons) were constructed by heating final amplified reactions from 60 to 95 °C for 15 s, 60 °C 30 s, 95 °C 15 s in single degree stepwise fashion. Primer efficiencies were calculated from readings derived from a standard curve of known DNA concentrations. Relative expression levels of target genes were calculated using the standardization as previously described by Pfaffl (2001). The GAPDH was used as a reference gene to standardize relative expression in the samples.

### Statistical analysis

All values are expressed as mean ± SEM. Analysis of variance was used to determine the significance of differences among the groups. Values of *P* < 0.05 were considered statistically significant.

## Results

### Growth of bovine embryonic stem cell-like cells on the different types of feeder layers

**Fig. 1:**
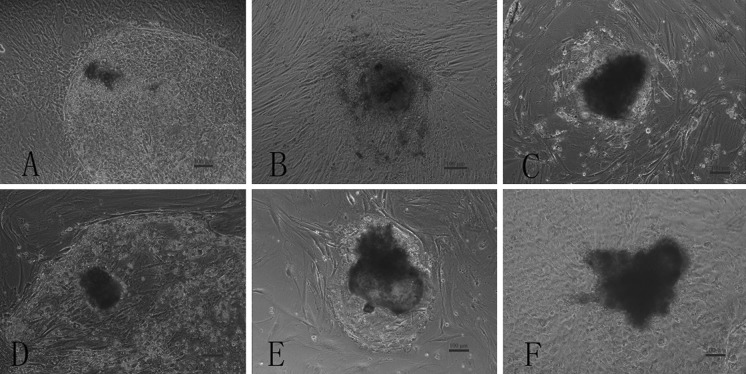
The clonal morphology of bovine embryonic stem cell-like cells in different feeder layers: mouse embryonic fibroblast feeder layers (**a**); bovine embryonic fibroblast feeder layers (**b**); mixed embryonic fibroblast feeder layer at a ratio of 1:1 (mouse embryonic fibroblast feeder layers: bovine embryonic fibroblast feeder layers) (**c**); mixed embryonic fibroblast feeder layers at a ratio of 2:1 (mouse embryonic fibroblast feeder layers: bovine embryonic fibroblast feeder layers) (**d**); mixed embryonic fibroblast feeder layers at a ratio of 1:2 (mouse embryonic fibroblast feeder layers: bovine embryonic fibroblast feeder layers) (**e**). STO cell feeder layers (**f**). Cells from the fifth passage are shown

The bovine embryonic stem cell-like cells were cultured on murine or bovine embryonic fibroblast feeder layers at the fifth passage, which had a clear border and apparent limitation to surrounding cells. There was no apparent eminentia, with the clone centers differentiating easily. Moreover, the colonies were thin and flat, and had not obvious bordering (Fig. 1a, b), while on the mixed feeder layer, bovine embryonic stem cell-like cells obviously grew to assembled cell masses and had clear colony borders. Moreover, bovine embryonic stem cell-like colonies were thick and solid. When mouse and bovine embryonic feeder layers were mixed together according to a ratio of 1:1, bovine embryonic stem cell-like cells obviously compacted to cell masses and had obvious boundaries with the feeder layer (Fig. 1c–e). The rate of attachment of blastocysts in the 1:1 ratio was significant higher (*P* < 0.05) than at the other ratios (Table 1). Similarly, the fifth passage of bovine embryonic stem cell-like cells on STO cell feeder layers were thick and closely connected to each other, with clear colony borders and obvious boundaries between the trophoblasts (Fig. 1f).

**Table 1:** Average attachment rate of different ratios of mixed layers (mouse embryonic fibroblasts feeder layers: bovine embryonic fibroblasts feeder layers)

	1:0	1:1	1:2	2:1	0:1
Number of blastocyst	84	87	95	82	85
Number of attached blastocyst	41	56	37	37	35
The rate of attachment blastocyst (%)	48.81 ± 7.4^b^	64.37 ± 15.5^a^	38.95 ± 14.0^c^	37.80 ± 11.0^c^	41.18 ± 6.3^b^

Values with different superscripts are significantly different. Each experiment was repeated 3 times. Data are expressed as mean ± SEM (n = 3)

^a^ *P* < 0.05, when the mixed ratio was set 1:1, the rate of attachment blastocyst was obviously higher than for the other ratios (*P* < 0.05)

### Differentiation of bovine embryonic stem cell-like cells in different feeder layers

**Fig. 2:**
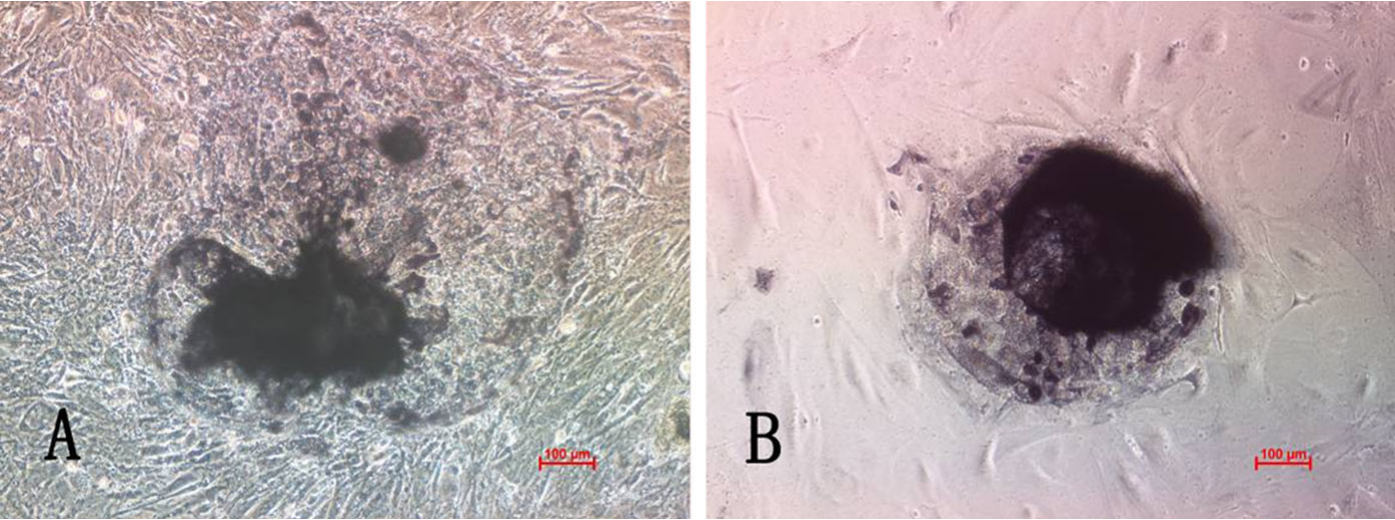
Alkaline phosphatase staining of bovine embryonic stem cell-like cells: mixed embryonic fibroblast feeder layers of 1:1 (**a**); STO cell feeder layers (**b**). Both of which were positive for AP staining. Cells from the tenth passage are shown

**Fig. 3:**
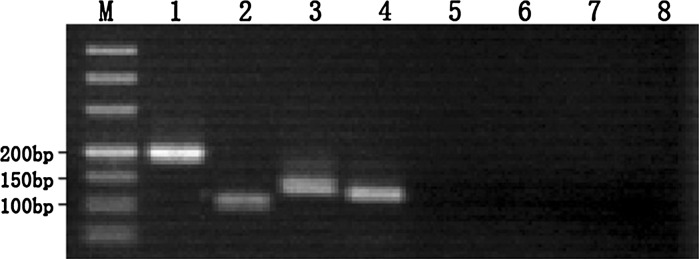
The RT-PCR analysis of bovine embryonic stem cell-like cells. OCT4, SOX2 and NANOG gene mRNA levels were observed in bovine embryonic stem cell-like cells cultured on mixed embryonic fibroblast feeder layers at 1:1 (similar results were obtained for bovine embryonic stem cell-like cells grown on STO cell feeder layers). Cells from the eighth passage are shown. *M* (50 bp) maker; *1* NANOG (195 bp); *2* OCT4 (106 bp); *3* SOX2 (121 bp); *4* GAPDH (128 bp); *5–8* negative control

**Fig. 4:**
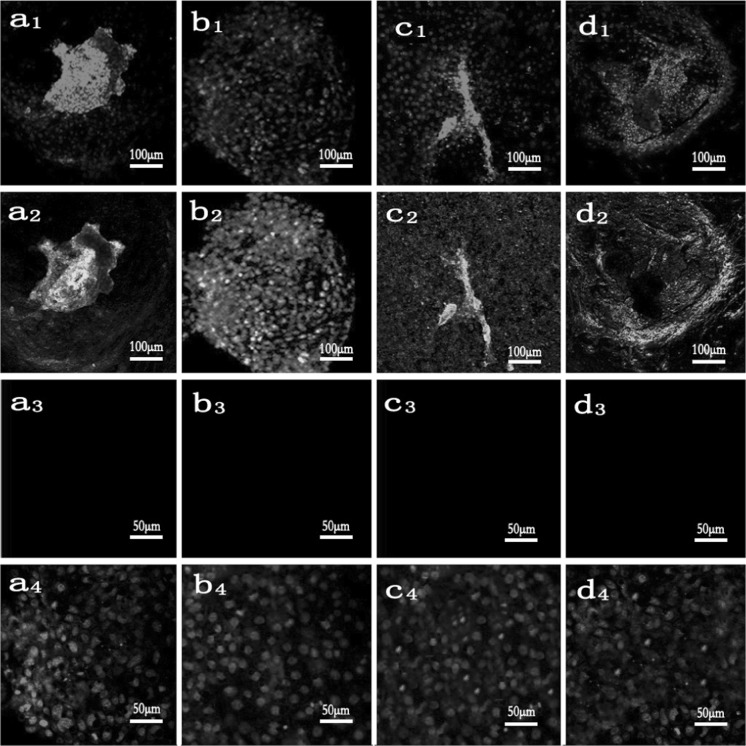
Representative immuonofluorescence images showing expression of OCT4, SSEA-1, and SSEA-4 in the bovine embryonic stem cell-like colony. Bovine embryonic stem cell-like colonies cultured on mixed embryonic fibroblast feeder layers were stained positively for OCT4 (**a2**) and SSEA-1 (**b2**); Bovine embryonic stem cell-like cells cultured on STO cell feeder layers were also stained positively for OCT4 (**c2**) and SSEA-4 (**d2**); negative controls for OCT4 (**a3**, **c3**), SSEA-4 (**d3**) and SSEA-1 (**b3**). All cells were stained with DAPI to highlight the nucleus (**a1**–**d1**), and negative controls were stained with DAPI (**a4**–**d4**). Cells from the tenth passage are shown

**Fig. 5:**
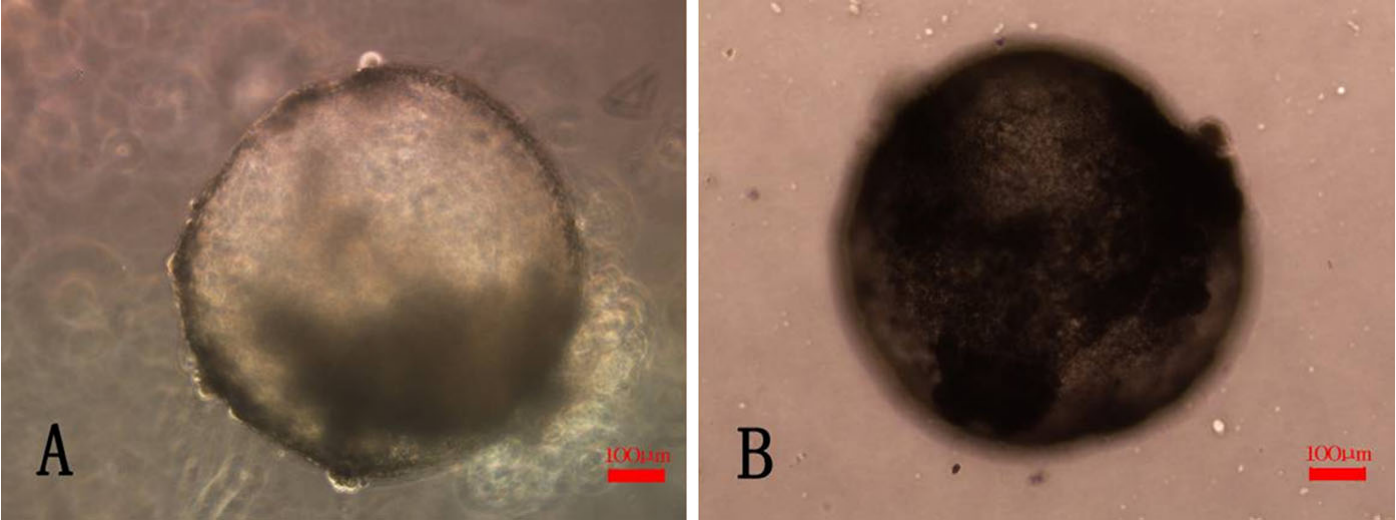
EBs differentiated in medium without leukemia inhibitory factor and feeder layer. Bovine embryonic stem cell-like cells had differentiated spontaneously into EBs on mixed embryonic fibroblast feeder layers (**a**) and STO cell feeder layers (**b**). Cells from the eighth passage are shown

Bovine embryonic stem cell-like colonies were classified as stem or differentiated colonies according to detection of undifferentiated state. On mixed embryonic fibroblast feeder layers and STO cell feeder layers, the tenth generation of bovine embryonic stem cell-like cells was respectively positive for ALP staining (Fig. 2a, b). The relative pluripotency-related levels of mRNA transcripts (expression levels) were monitored in bovine embryonic stem cell-like cells by using RT-PCR. The results showed that OCT4, SOX2 and NANOG were expressed in bovine embryonic stem cell-like cells on mixed embryonic fibroblast feeder layers at 1:1 and STO cell feeder layers. Cells from the eighth passage are shown (Fig. 3). Furthermore, bovine embryonic stem cell-like colonies on mixed embryonic fibroblast feeder layers at the tenth passage strongly stained positive for OCT4 (Fig. 4a) and SSEA-1 (Fig. 4b). Similarly, OCT4 (Fig. 4c) and SSEA-4 (Fig. 4d) were also strongly stained in undifferentiated bovine embryonic stem cell-like cells on STO cell feeder layers at the tenth passage. When cultured for 7–10 days in medium without LIF, the eighth generation of bovine embryonic stem cell-like cells on mixed embryonic fibroblast feeder layers and STO cell feeder layers, respectively, spontaneously differentiated into EBs (Fig. 5a, b).

### Pluripotency-related gene expression in mouse embryonic fibroblast feeder layers

**Fig. 6:**
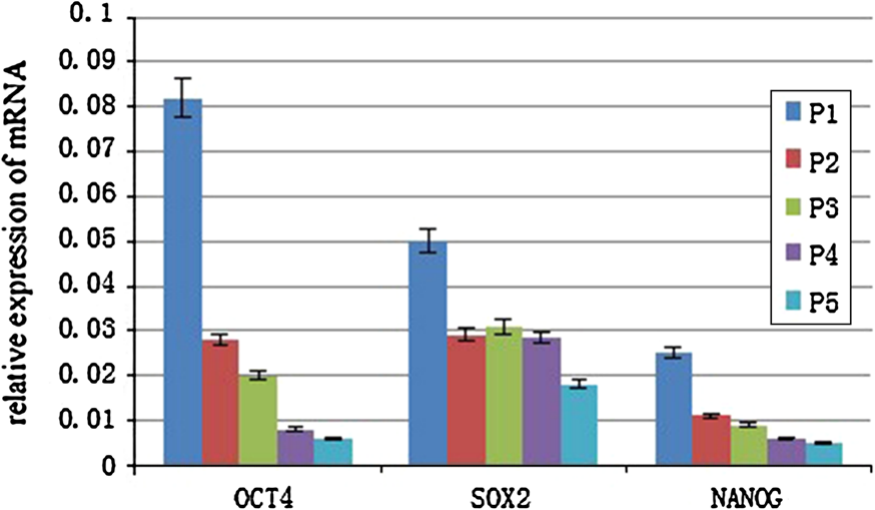
Relative expression of the major pluripotency-related transcription factors in bovine embryonic stem cell-like cells cultured on murine embryonic fibroblast feeder layers. Expression of OCT4, SOX2 and NANOG were significantly lower in the fifth passage than in the first passage. Cells from the first passage to fifth passage are shown

Bovine embryonic stem cell-like colonies on mouse embryonic fibroblast feeder layers at passages 1–5 were individually collected and mRNA transcript levels were determined by quantitative RT-PCR. The data showed that the OCT4, SPX2, and NANOG levels were found to be significantly reduced in the fifth passage of bovine embryonic stem cell-like cells compared to those in the first passage (Fig. 6).

### Comparison of pluripotency-related gene expression in three feeder layers

**Fig. 7:**
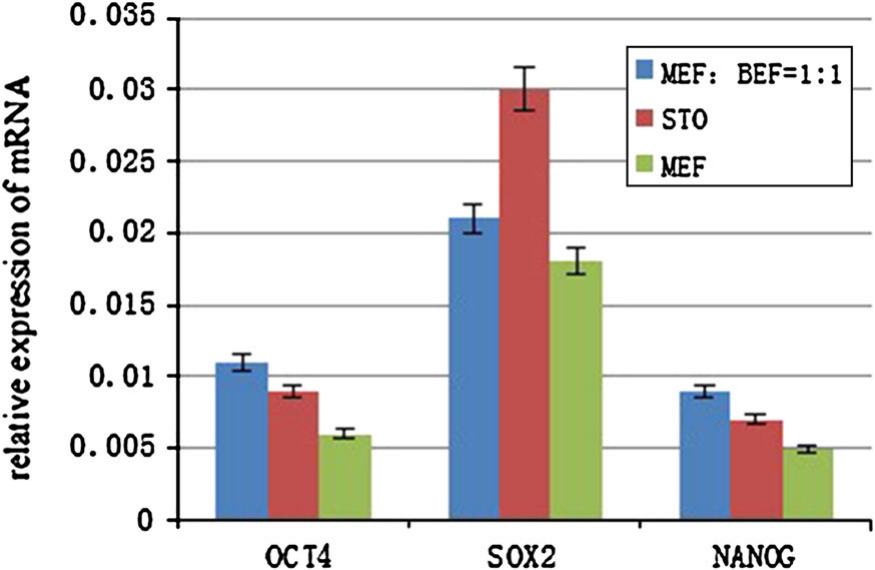
Comparison of OCT4, SOX2, and NANOG expression in bovine embryonic stem cell-like cells cultured on murine embryonic fibroblast feeder layers, STO cell feeder layers and mixed embryonic fibroblast feeder layers. OCT4, SOX2, and NANOG expression in bovine embryonic stem cell-like cells was significantly higher when cultured on STO cell feeder layers and mixed embryonic fibroblast feeder layers than on murine embryonic fibroblast feeder layers. Cells from the tenth passage are shown

The tenth generation of bovine embryonic stem cell-like cells on mixed embryonic fibroblast feeder layers, including mouse fibroblast cell line feeder layers and mouse embryonic fibroblast feeder layers, mouse embryonic feeder layers and STO cell feeder layers were individually collected and mRNA transcript levels were determined by quantitative RT-PCR. The results demonstrated that OCT4, SOX2, and NANOG were expressed at significantly higher levels on mixed embryonic fibroblast feeder layers and STO cell feeder layers than on mouse embryonic feeder layers (Fig. 7).

## Discussion

The derivation and maintenance of embryonic stem cell lines from bovine blastocysts is perhaps the most difficult and challenging among all mammals (Gong et al. 2010). Therefore, improvements are needed in order to supply defined feeder cells to bovine embryonic stem cells culture. In the present study, we demonstrated that OCT4, SOX2, and NANOG levels were found to be significantly lower in the fifth passage of bovine embryonic stem cell-like cells than in the first passage, when bovine embryonic stem cell-line cells were cultured on murine embryonic fibroblast feeder layers at 1–5 passages. However, recent findings identifying high levels of OCT4, SOX2, and NANOG are associated with pluripotency and self-renewal, while low levels are associated with a tendency to differentiation (Avilion et al. 2003; Pesce and Schöler 2001; Miyanari and Torres-Padilla 2012). This may be the reason that bovine embryonic stem cell-like cells cannot continue to grow and to be passaged. Obviously, it is important to look for a new feeder layer system which could sustain the pluripotency and undifferentiated state of bovine embryonic stem cell-like cells. We have shown that the morphology of bovine embryonic stem cell-like cells on mixed embryonic fibroblast feeder layers was obvious better than when culturing on single embryonic fibroblast feeder layers. The reasons for this may be that mouse embryonic fibroblast feeder layers have a shorter growth time and stronger growth, and secrete factors such as LIF that promote embryonic stem cell growth when used as feeder layers (Jin et al. 2012). Mouse embryonic fibroblast feeder layers also could widely provide space for growth. Meanwhile, bovine embryonic fibroblast feeder layers as homologous feeder layers easily fused with trophoblast so as to effectively prevent embryonic stem cells transformation to trophoblast stem cells (Talbot and Powell 1995). Owing to the molecule affinity on both embryonic stem cells and fibroblasts surface, embryonic stem cells can easily attach onto feeder cells. Therefore, the mouse embryonic fibroblast feeder layers in common with bovine embryonic fibroblast feeder layers could mutually promote the adherence of bovine embryos and inhibit the differentiation of bovine embryonic stem cell-like cells. Furthermore, mouse embryonic fibroblast feeder layers and bovine embryonic fibroblast feeder layers at a ratio of 1:1 was superior not only with respect to morphology of the cells, but also to the higher adherence rate of bovine embryos. In addition, we also found that the two embryonic fibroblast at this ratio could be cultivated to the fifteenth generation at least, whereas at the ratio of 1:2 or 2:1, they could be only cultivated to the sixth and eighth generations, respectively, and the cells had differentiated. Thus, the mixed embryonic fibroblast feeder layers at ratio of 1:1 might better support in vitro subculture of bovine embryonic stem cell-like cells to acquire excellent colony form compared to mouse or bovine embryonic fibroblast feeder layers. Apparently, the mixed embryonic fibroblast feeder layers could completely replace the routine mouse or bovine embryonic fibroblast feeder layers.

In this study, the bovine embryonic stem cell-like cells on STO cell feeder layers grew in assembled cell masses and had clear colony borders. The cells were dense and circular, which had an obvious boundary with trophoblast layers. Two or three days after subculturing, the primary colony formation rate was around 47 %, which is within the range of reported colony formation (13–67 %) (Stice et al. 1996; Strelchenko 1996; Park et al. 2003; Gong et al. 2010). Recently, it has been suggested that STO cells as feeder layers could promote the adherence of the embryonic stem cells and could also secrete a variety of factors which can maintain the undifferentiation state of embryonic stem cells, such as leukemia inhibitory factor or basic fibroblast growth factor (Talbot and Powell 1995; Park et al. 2004). This may be the reason why bovine embryonic stem cell-like cells have a better morphology and higher primary colony formation rate on STO cell feeder layers. When STO cells were prepared as feeder layers, we also found that the morphology of STO cells reached the optimal growth stage at the fifth passage. Less than ten passages of STO cells are considered as optimal for their use as feeder layers. Originally, with long-term culture and passage, trypsin and the toxic effects of mitomycin had a side effect on STO cells even when trypsin and mitomycin were washed out. The changes that included proliferation, morphology and viability of STO cells affected the quality of the STO cells as a feeder layer for embryonic stem cell growth. Compared with mouse embryonic fibroblast cells, STO cells could be cultured long-term in vitro. STO cell is widely and conveniently used for culturing hybridoma cells, pluripotent teratocarcinoma cells and a variety of animal pluripotent stem cells (Chen et al. 1999; Martin et al. 1977; Lim and Bodnar 2002; Martin and Evans 1975). Moreover, bovine embryonic stem cell-like cells were identified that had the characteristics of stem cells. Therefore, STO cell feeder layers have the potential to support the establishment and maintenance of bovine embryonic stem cell-like cells.

We also demonstrated that OCT4, SOX2, and NANOG were expressed at significantly higher levels in bovine embryonic stem cell-like cells cultured on mixed embryonic fibroblast feeder layers and STO cell feeder layers than on mouse embryonic fibroblast feeder layers. This result implies that the mixed embryonic fibroblast feeder layers and STO cell feeder layers are superior in regulation of self-renewal and differentiation of bovine embryonic stem cell-like cells than mouse embryonic fibroblast feeder layers. Therefore, the STO cell feeder layers and mixed embryonic fibroblast feeder layers are more suitable than murine embryonic fibroblast feeder layers for the culture of bovine embryonic stem cell-like cells.

In conclusion, our study strongly suggests that mixed embryonic fibroblast feeder layers and STO cell feeder layers can be used as valuable candidate feeder layers to support the cultivation and isolation for bovine embryonic stem cell-like cells.
